# Recent Developments and Advancements in Graphene-Based Technologies for Oil Spill Cleanup and Oil–Water Separation Processes

**DOI:** 10.3390/nano12010087

**Published:** 2021-12-29

**Authors:** Salma Elhenawy, Majeda Khraisheh, Fares AlMomani, Mohammad K. Hassan, Mohammad A. Al-Ghouti, Rengaraj Selvaraj

**Affiliations:** 1 Department of Chemical Engineering, College of Engineering, Qatar University, Doha 2713, Qatar; se1105821@student.qu.edu.qa (S.E.); falmomani@qu.edu.qa (F.A.); 2 Center of Advanced Material (CAM), Qatar University, Doha 2713, Qatar; mohamed.hassan@qu.edu.qa; 3 Environmental Science Program, Department of Biological and Environmental Sciences, College of Arts and Sciences, Qatar University, Doha 2713, Qatar; mohammad.alghouti@qu.edu.qa; 4 Department of Chemistry, College of Science, Sultan Qaboos University, Muscat 123, Oman; rengaraj@squ.edu.om

**Keywords:** nanomaterials, graphene, water–oil separation, graphene oxide, reduced graphene oxide, foams, sponges, metal meshes

## Abstract

The vast demand for petroleum industry products led to the increased production of oily wastewaters and has led to many possible separation technologies. In addition to production-related oily wastewater, direct oil spills are associated with detrimental effects on the local ecosystems. Accordingly, this review paper aims to tackle the oil spill cleanup issue as well as water separation by providing a wide range of graphene-based technologies. These include graphene-based membranes; graphene sponges; graphene-decorated meshes; graphene hydrogels; graphene aerogels; graphene foam; and graphene-coated cotton. Sponges and aerogels modified by graphene and reduced graphene oxide demonstrated effective oil water separation owing to their superhydrophobic/superoleophilic properties. In addition, oil particles are intercepted while allowing water molecules to penetrate the graphene-oxide-coated metal meshes and membranes thanks to their superhydrophilic/underwater superoleophobic properties. Finally, we offer future perspectives on oil water separation that are hindering the advancements of such technologies and their large-scale applications.

## 1. Introduction

Oily wastewaters, oil spills from the petroleum industry, and oil-shipping accidents have caused detrimental effects on aquatic ecosystems [1,2,3,4,5,6]. Such releases can occur from various sources including oilrigs, transporters, and underwater pipelines.

Several methods were proposed by various number of studies to tackle oil spillage cleanups; these methods include absorption [7,8,9], dispersants [10,11,12,13,14,15,16,17,18,19,20], solidifiers [21,22,23], and controlled burning [24]. Absorption is one of the most commonly used methods in the separation and recovery of spilled oil from water thanks to its good efficiency, simple operation, and flexibility to combine with other methods. Oil absorbents should have unique characteristics such as high oil sorption capacity, fast oil sorption rate, low density, low cost, and reusability. Recently, a wide variety of advanced and novel absorbent materials possessing oleophilic and hydrophobic surfaces with excellent adsorption performance have been synthesized for the separation and recovery of oil from water. Among such absorbents are fiber sheets [25,26,27,28,29], alumina [30,31], zeolites [32,33,34], silica [35], polypropylene [36,37,38,39], and polystyrene [40,41,42,43]. Such sorbents have certain disadvantages that limit their absorption efficiency, including their low sorption capacities, low recovery efficiencies, production of toxic wastes, and poor buoyancy characteristics. The development of lightweight materials with high sorption capacity, environmental friendliness, and chemical inertness is crucial for oil-spill response operations.

One of the most critical issues related to the oil–water separation is surface wettability. The control of such wettability can allow one phase permeation and repel the other phase [44]. Accordingly, water/oil separation materials or membranes are generally divided into two categories: super hydrophobic/superhydrophilic membranes and hydrophilic/underwater superhydrophobic. The latter is oil repellent and permeable to water. This type is best applied for light oil/water separations. On the other hand, and in the case of the superhydrophobicity materials and membranes, heavy oil/water applications and separations are possible. In the case of unidirectional separation of light oil/water mixtures, it is reported that light oil molecules will be trapped between the water layer and the water repellent membranes. As a result, the importance of inverse wettability membranes was highlighted for effective and continuous oil/water separation [44]. The fabrication of such membranes was achieved by coating a number of materials such as metal meshes, sponges, polymers, metal oxides, and many others. Chemical and electrical depositions, in addition to hydrothermal reaction pathways, were reported with many challenges ranging from the need for specialized equipment, to the use of toxic reagents and time-consuming procedures [44]. Accordingly, and in an effort to overcome such complexity, graphene and its derivatives have been vastly investigated [44,45,46,47,48].

Graphene [49] has the basic structural element of carbon allotropes, including graphite, fullerenes, and carbon nanotubes [50]. Graphene has a single layer of carbon atoms arranged in one plane [51]. The structure of graphene is a single planar sheet of sp^2^ carbon–carbon bonded atoms that are densely packed into a honeycomb crystal-like lattice [52]. Graphene possesses high tensile strength, which is estimated to be 300 times stronger than A36 structural steel and 40 times stronger than diamond [53]. Additionally, graphene has good electronic properties thanks to the bonding and antibonding of the pi orbitals. Graphene shows an extraordinary thermal, electrical, and physical properties [54]; Table 1 displays some of the physical properties of graphene. Graphene has a large surface area, with a high chemical and thermal stability. Graphene is a superb new class of material whose remarkable properties made it the subject of ongoing studies in many fields [55]. Graphene is an excellent adsorption material and has several applications in the field of oil spill cleanup and water purification processes [56].

**Figure 1:**
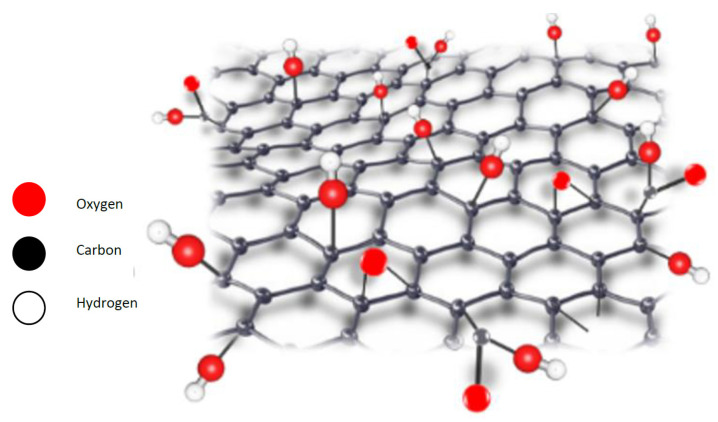
A representation of the 3D structure of GO.

One of the widely explored graphene-based materials is graphene oxide (GO), produced by the direct oxidation of graphite. GO exhibits attractive chemical, electrical, and optical properties caused by the graphene skeleton and its oxygen content (Figure 1). The oxygenated functional groups located on the edges of GO sheets cause its hydrophilic properties and make the surface modifications easier, leading to the creation of other graphene-based materials. Because of this structure and the large amounts of oxygen-containing groups on its surface and edges, enhanced stability in water and other polar solvents results. This leads to the enhancement of the hydrophilicity of the coated surface. Accordingly, GO offers great potential for the preparation cost-effective, large-scale production of graphene-based materials that can be used for many applications. If the oxygenated functional groups are eliminated, the graphene can be reduced to form reduced graphene oxide (rGO). The GO and its derivatives such as rGO are used to enhance the material wettability of sponges, metal meshes, and aerogels, which results in superhydrophilic/underwater superoleophobic materials [47].

Owing to the detrimental effects of oil spills on the aquatic ecosystem and the unique properties of graphene materials, this review article covers several graphene-based technologies for oil/water separation processes. These technologies include graphene-based membranes, graphene sponges, graphene decorated meshes, graphene hydrogels, graphene aerogels, graphene foam, and graphene-coated cotton, hoping to provide the world with efficient and environmentally friendly approaches to remove the detrimental oil from water.

## 2. Oil–Water Separation Techniques

Typically, an oil water separator consists of three main segments: 1—a filter unit, 2—separator unit, and 3—an oil content monitor and control unit. The filter and separator units are considered as the main treatment units, where various designs and principles are applied. The centrifugal and gravity separators are commonly used in the first treatment stage, followed by other separation techniques, which is known as polishing treatment. The polishing unit includes the following: absorption, adsorption, flotation, flocculation and coagulation, and biological treatment. The choice of the oil/water separation technique is crucial for an efficient separation process.

**Figure 2:**
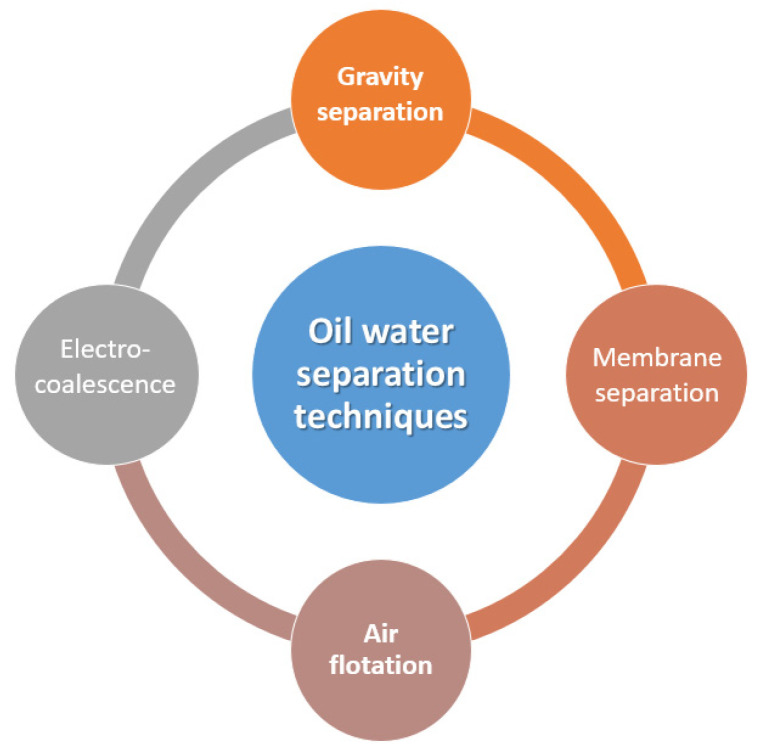
Principal oil/water separation techniques.

The traditional and most frequently used methods for oil/water mixture separation are shown in Figure 2. However, the applications of such methods are limited with their drawbacks such as low separation efficiency, high cost, and secondary pollution. As a result, research studies were conducted to find more suitable methods that overcome these drawbacks. Among the mentioned separation methods, gravitational separation has merits in terms of treated volume; however, it is inefficient in the isolation of emulsified mixtures. Moreover, membrane filtration techniques are considered the most efficient method in the treatment of oil/water mixtures owing to their simple operational process, but their efficiency is greatly limited as the separation of stable emulsions requires small-sized pores. In addition, membranes are limited with their high cost, fouling, and low recyclability, which narrow their application in emulsion separation. Thus, the development of novel materials and methods with superior selectivity, excellent stability, and high separation efficiency became crucial for an effective separation of oil/water mixtures [66].

## 3. Graphene-Based Membranes in Oil/Water Separation

**Figure 3:**
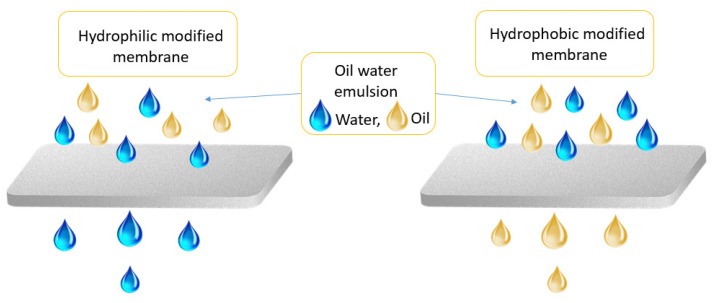
Schematic diagram of oil/water membrane separation process.

Separation of oil from water emulsions remains as a serious issue because of the high discharge volume of oily wastewater from industrial processes. Membrane technology has been widely used in oil/water separation owing to its high separation efficiency and simple operational process (Figure 3). However, the membrane hydrophobic properties can lead to the membrane fouling phenomenon, which shortens the membrane’s life. Consequently, modification of the membrane, specifically hydrophilicity enhancement, is crucial to optimize the performance of the membrane. Over the past years, graphene and its derivatives, specifically graphene oxide (GO), has captured the attention of many researchers in the field of membranes for water treatment owing to its superb thinness and distinguishable layered structure. These unique features increase the permeation fluxes and enhance the hydrophilic and physicochemical properties, making it excellent for oil/water separation [67]. Table 2 below shows the performance of several graphene-based membranes in oil/water mixtures.

Singhal, George [74] developed a novel oleophobic and super-hydrophilic graphene-based membrane using a cost-effective and simple vacuum filtration methodology. Before developing the membranes, the authors crosslinked the graphene oxide (GO) sheets with the tannic acid (TA) molecules to improve their surface and mechanical properties. The results of this study showed that the TA crosslinked GO membranes are very efficient in treating oily waste water released from industries [74]. Abdalla, Wahab [75] fabricated polysulfone (PS) mixed matrix membranes (MMMs) using a phase inversion method that contains aspartic acid (AA) functionalized graphene oxide (fGO) with enhanced oil rejection, flux, and resistance to fouling. The results of the authors’ study showed an increase in the membrane’s oil rejection, water permeability, and hydrophilicity, at very low fGO loadings. Hence, PS/fGO MMMs can be successfully used in oil/water separation processes [75].

The treatment of wastewater discharged from the oil and gas industry presents a serious issue because the wastewater contains a complex mixture of oil and water that is difficult to treat. Thus, an efficient separation process of oily wastewater is in great demand and is still considered a worldwide challenge. Alammar, Park [76] developed graphene oxide (GO), reduced GO (rGO), and polybenzimidazole (PBI) nanocomposite membranes using the phase inversion and common blade coating technique for treating water produced from the oil and gas industry. The nanocomposite membranes were dip-coated by polydopamine (PDA), with excellent antifouling properties. In addition, the incorporation of GO into the PBI matrix has resulted in a superior oil-removal efficiency, reaching 99.9%. Furthermore, the addition of GO has increased the membranes’ mechanical stability. The nanocomposite membranes provided are very promising, and are highly efficient membranes for oil-in-water emulsion separation with a high capability of de-oiling high salinity emulsions under harsh industrial conditions [76]. Zhang, Xiao [77] fabricated a superoleophilic and under oil superhydrophobic poly (vinylidene fluoride) (PVDF)/graphene (GE) composite nanofibrous membrane (GPNM) via a simple one-step electrospinning method for gravity-driven surfactant-stabilized water-in-oil emulsions separation. The synthesized composite membrane revealed an outstanding water-in-oil emulsions separation efficiency, reaching 99.8%. These results show that the composite nanofibrous membrane shows great promise in the remediation of wastewater from oil [77]. Cheng, Barras [78] have successfully prepared a superoleophilic/superhydrophobic reduced graphene oxide-polydopamine functionalized with 1H,1H,2H,2H-perfluorodecanethiol (rGO-PDA-PFDT) membrane via a simple and facile two-step method. The rGO-PDA-PFDT membrane was applied for oil/water separation. The membrane was capable of separating chloroform from water owing to its superhydrophobicity and superoleophilicity. Hence, the synthesized rGO-PDA-PFDT membrane is very promising in oil/water separation processes [78]. Shao, Yu [79] have successfully synthesized a sepiolite/graphene oxide (Sep/GO) membrane for wastewater treatment purposes. The synthesized Sep/GO membranes were very effective in separating oil-in-water emulsions as a result of their high oil/water selectivity and low oil adhesion. Furthermore, in harsh environments, the membrane maintained an underwater superior stability and superoleophobicity. Hence, the Sep/GO membrane is an efficient membrane in oil/water separation and wastewater treatment processes [79].

Water pollution by organic and inorganic contaminants has increased the global attention on wastewater treatment and renewable industries. A wide range of materials have been synthesized for the remediation of pollutants from water; however, materials that are capable of soluble-contaminant degradation and emulsion separation in a single step are rare. Qian, Chen [70] reported a TiO_2_/sulfonated graphene oxide/Ag nanoparticle (TSA) membrane, which holds useful photocatalytic and wettability properties. The synthesized membrane exhibited an efficient oil/water separation performance, and excellent photodegradation under ultraviolet irradiation of solubilized methylene blue (MB). Thus, the membrane has promising applications in oil/water separation and wastewater treatment [70]. Liu, Wu [80] prepared a highly stable graphene oxide (GO) membrane via a simple coating technique for oil-in-water emulsions’ separation. The authors have used a mussel inspired polydopamine (PDA) as a covalent linker, and have successfully modified the mixed cellulose ester membrane (MCEM) with a PDA layer via self-polymerization, then the GO nanosheets were attached to PDA/MCEM using a simple vacuum filtration method, leading to the formation of GO/PDA/MCEM. GO/PDA/MCEM was then used to separate oil-in-water emulsions. The results of this study show that the prepared GO/PDA/MCEM is very effective in the separation of oil-in-water emulsions. Thus, this study provides a simple, economical, and feasible way for the preparation of a highly stable and efficient GO-based membrane for oil-in-water emulsions’ separation [80]. Peng et al. [81] have successfully fabricated a RGO@SiO_2_ nanohybrid, and cooperated with dopamine to form novel PVDF/RGO@SiO_2_/PDA nanohybrid membranes using a surface deposition method. The novel membranes were successfully used for the remediation of oils and cationic dye from wastewater. Hence, the synthesized PVDF/RGO@SiO_2_/PDA composite membranes have a promising potential as advanced separation membranes for water purification [81]. Pollution of oil-in-water and removal of oil from water are gaining great attention owing to the increase in environmental issues. Membrane technology is one of the main effective solutions for oil/water separation. However, there are limitations in using polymeric membranes for oil/water separation owing to its surface properties, mechanical, and thermal properties. Prince et al. [82] have demonstrated a simple method to increase the hydrophilicity of a polyethersulfone (PES) hollow fiber ultrafiltration (UF) membrane using hydroxyl, carboxyl, and amine modified graphene attached to poly acrylonitrile-co-maleimide (G-PANCMI). The developed membranes by the authors were characterized for its water and oil contact angle, morphology, water permeability, and liquid entry pressure of oil (LEP_oil_), and subjected to a filtration test of oil emulsion in water. The results of this study show that the synthesized PES-G-PANCMI membrane is very promising in oil/water separation [82]. Zhang et al. [39] synthesized polyacrylonitrile/graphene oxide (PAN/GO) composite fibers with a spindle-knot structure via facile electrospinning, and then hydrolyzed (H-PAN/GO) for enhancing their chemical features, and their oil/water separation performance was evaluated. The fabricated membrane exhibited an outstanding oil/water separation performance owing to the hydrolyzed PAN chemical features and the spindle-knotted structure induced by GO. The H-PAN/GO fibrous membrane offers a new insight into the fabrication of next generation membrane for separating oil/water emulsion [83]. Hu et al. [84] synthesized a novel membrane material via graphene oxide (GO) modification on the commercial 19-channel Al_2_O_3_ ceramic microfiltration membrane, in order to obtain high oil rejection in the oil/water emulsion microfiltration. The results of the study show that the modified membrane has higher oil rejection compared with the unmodified membrane. Hence, modification with graphene oxide plays a crucial role in oil/water separation performance [84]. A recent review [85] evaluated the performance of GO-based membranes. The study highlighted the importance of this class of membranes with respect to enhanced fouling resistance, solute selectivity, and solution flux compared with unmodified membranes. It was highlighted that the addition of GO on the surface of the membrane altered its morphology, surface wettability, and the nature of the functional groups.

As such, and considering the large volume of published work on the use of GO enhanced membranes, the practical use of this technology is still at laboratory scale application and requires advancements to reach a pilot or a larger scale application. Some of the main challenges associated with the use of GO or GO-based composite membranes for such applications are related to the proper decomposition of the nanoparticles onto the surface of the membranes. To this end, further studies are still required to evaluate the impact of the ratio of GO-based nanoparticles on the membrane matrix. Optimization of the concentration and distribution of the graphene-based materials is required to achieve the best performance of membranes. In addition, the biggest challenge for successful application is the large-scale fabrication of the GO composite membranes. Overcoming such challenges may pave the way to effective application for oil/water separations and other water treatment applications.

### 3.1. Graphene-Based Sponges for Oil/Water Separation

**Figure 4:**
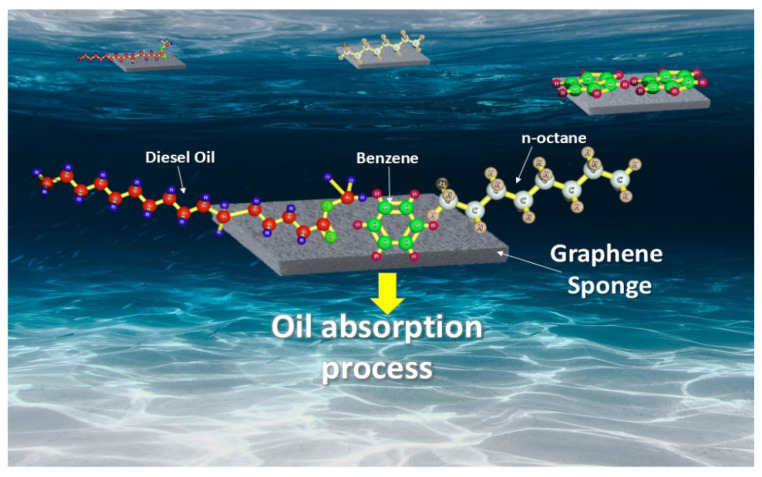
Crude oil derivatives’ absorption via graphene sponge in seawater.

Based on several studies, three-dimensional (3D) porous sponges are very promising high-capacity sorbents owing to their large surface area. However, most of the commercially available sponges are poorly hydrophobic, which limits their application for oil spill cleanup and recovery [86]. Thus, surface modification is a necessity to increase the hydrophobicity and decrease the water-wettability of the sponges to obtain the desired superoleophilic and superhydrophobic oil sorbent. Sponges are usually coated with graphene, carbon nanotubes, polypyrrole, polyaniline nanofibers, and nanocrystals to improve their superoleophilicity and superhydrophobicity [87]. The graphene-based sponge exhibits high sorption capacities for several types of oils owing to its high surface area [88] (Figure 4). Typically, carbon materials have shown some attractive properties, such as mechanical integrity, high capacity, high porosity, and large pore volume [89]. Great efforts have been devoted to optimizing and developing carbon materials for oil absorption [90], with more hydrophobicity, oleophilicity, functional properties, and specific thermal property [91]. Table 3 shows the sorption capacities of several graphene-based modified sponges for various types of oils in water.

With the industrial revolution and the growth in industrial activities, large amounts of oil discharges are released into the aquatic environment. Thus, superhydrophobic and superoleophilic materials with reusability are needed.

Polyurethane (PU) sponge has attracted great attention in the oil/water separation field for its distinguishable three-dimensional (3D) porous structure. On the other hand, its oil-absorption efficiency is significantly hindered by its poor hydrophobicity. Rahmani, Samadi [8] investigated the application of graphene-coated polyurethane (PU) sponge for the oil spills’ selective absorption from water. The authors have integrated nanoporous graphene (NPG) and graphene oxide (GO) flakes with the porous structure of PU, leading to the preparation of NPG/PU and GO/PU, respectively. The results of the study show that the NPG/PU sponges had a high capacity in the removal of crude oil and organic liquid stains. The absorption capacity for NPG/PU sponge was greater than that of GO/PU sponge. Thus, in oil spill remediation, graphene-coated polyurethane (PU) sponge is an efficient and cost-effective method [8]. Xia et al. [99] used a one-pot solvothermal method to facilely fabricate a superhydrophobic reduced graphene oxide-coated polyurethane (RGO@PU) sponge in the presence of ethanol. The RGO@PU sponge with a superhydrophobic surface prepared by the authors exhibited a high oil-absorption capacity, 37 times greater than its original mass. The results of the study prove that the RGO@PU sponges have great potential as an absorbent for oil/water separation owing to their rapid oil-absorption rate and high oil/water separation efficiency (~99%) [99]. Qiang et al. [102] synthesized graphene oxide nanoribbons (GONRs) functionalized by silane molecules with several hydrophobic end-groups, and fabricated silane functionalized reduced GONR (silane-f-rGONR) coated polyurethane (PU) sponge composites using a facile dip-coating process. The results of the study show that the two types of silane modified sponges demonstrated distinct super-hydrophobicity and tunable oleophilicity/oleophobicity. The porous silane-f-rGONR@PU composites exhibited a superb selectivity for oil-water separation and high oil/solvent absorption capacity at static state. In addition at the dynamic shaking state, the porous silane-f-rGONR@PU composites have shown a superb oil/water separation efficiency compared with the rGONR@PU composite with poor oil/water separation. This study has shown a new method for synthesizing the super-hydrophobic, mechanically flexible and electrically conductive porous rGONR-based composites, with a promising application in oil pollution remediation from water [102]. Khalilifrad et al. [103] synthesized a modified magnetic superhydrophobic polyurethane sponge by connecting graphene oxide (GO), coated with functionalized oleic acid Fe_3_O_4_ nanoparticles on three-dimensional microstructure of commercial polyurethane sponge (Fe_3_O_4_@OA@GO-PU) using a low-cost and simple dip coating method. The results of the study showed that the eco-friendly modified sponge has efficiently adsorbed various oils from water with a high adsorption capacity. Consequently, the synthesized modified sponge in this study is a superb adsorbent for efficient applications in the separation of oil from water [103].

Crude oil spill cleanup and recycling oily wastewater are of great concern globally. Qin et al. [104] developed a simple approach to synthesize a solar-driven superhydrophobic/oleophilic polybenzoxazine/reduced graphene oxide wrapped-cellulose sponge (PBZRGOS) for an effective oil/water separation process and rapid crude oil remediation from water. Polybenzoxazine (PBZ) was introduced into the cellulose sponge as a binder to provide low energy for the surface. The results of the study show that the modified sponge exhibited a high oil/water separation efficiency, reaching 99.1%, which surpasses other reported modified sponges. Hence, it can be concluded that the synthesized superhydrophobic cellulose sponge can be successfully applied as a competitive functional sponge for the removal of spilled crude oil from water [104]. Pethsangave et al. [89] synthesized a novel superhydrophobic ultra-light graphene-based carrageenan sponge (GCS) absorbent via one pot hydrothermal method, in order to selectively adsorb oils and organic solvents from water mixtures. In the presence of formaldehyde, the graphene oxide (GO) nanosheets were reacted by the insertion of carrageenan, producing hydrophobic cross-linked structure between them. The results of the study show that the GCS had an excellent oil sorption capacity within the range of 25.2 to 50 g of oil per gram of adsorbent. The present study suggests that the synthesized carrageenan sponge (GCS) has great potentials in the removal of oil from water [89].

The development of porous sponge materials with surface super-hydrophobicity and high mechanical robustness is strongly required for several types of applications. The main drawback is the realization of the multiple functionalities simultaneously using a facile approach. Moa et al. [105] fabricated a super-hydrophobic, flame-retardant, and mechanically flexible functionalized silica/graphene oxide wide ribbon (GOWR) coated melamine sponge (M-GOWR@MF) via a simple two-step surface-modifying method. The results of the study show that the as-prepared MF sponge composites demonstrated super-hydrophobicity/super-oleophilicity and high mechanical robustness, leading to a superb absorption capacity for both heavy and floating oils from water and efficient performance of continuous oil/water separation. Thus, the hybrid functionalized silica/GOWR network in this study provides a new approach to develop advanced multi-functional polymer sponge composites for oil removal purposes from water [105]. Jamsaz et al. [100] investigated a novel recyclable, superoleophilic, superhydrophobic, and flame-retardant graphene-based polyurethane (PU) sponge fabricated by functionalizing polyurethane (PU) with reduced graphene oxide (RGO) and orthoaminophenol (OAP). The synthesized RGO/OAP/PU sponge in the study showed a high sorption capacity for a wide range of oils. Thus, the RGO/OAP/PU sponge can be successfully applied in the field of oil/water separation [100]. A superoleophilic/superhydrophobic material was prepared by coating hexadecyltrimethoxysilane-grafted reduced graphene oxide on a melamine sponge skeleton (HDTMS/rGO-MF) using a low-cost and simple method to absorb various oils and organic solvents from water [106]. The results of the study show that the absorption capacities of HDTMS/rGO-MF for several oils and organic solvents were 8.79–20.85 g/g. Several oil spill accidents have caused catastrophic environmental issues. Hence, hydrophobic sorbents have gained great attention for oil spill remediation. Zhou et al. [107] developed an oil/water separation material with robust mechanical properties by the modification of modifying melamine sponge with silk fibroin-graphene oxide (SGMS). The authors have used the silk fibroin as a molecular binder to combine melamine sponge and graphene oxide. After modification, the sponge surface became hydrophobic. The results of the study have shown that the SGMS exhibited an efficient oil/water separation, excellent oil adsorption capacity, good mechanical properties, and superior recyclability. These superb properties show that SGMS can be successfully applied in the adsorption of oil and organic pollutants under realistic conditions [107]. Wang et al. [108] developed a solar-driven self-heated sponge as a novel sorbent to achieve rapid collection of crude oil from spills using the advantage of light-to-heat conversion to reduce the oil viscosity significantly. The authors have fabricated the sorbent via facile dip-coating of reduced graphene oxide on a commercial melamine sponge. The sponge produced in this study has shown a distinguishable remediation of viscous crude oil spills [108].

The mechanical recovery of oils via oil sorbents is one of the most important and crucial methods in managing marine oil spills. However, the properties of the oil released into sea are affected by external environmental conditions. Shiu et al. [88] demonstrated a graphene-based (GB) sponge as a novel sorbent for the removal of crude oil and compared its performance with a commercial sorbent sheet under several environmental parameters. The results of this study showed that the GB sponge demonstrated excellent superoleophilic and superhydrophobic characteristics. Hence, the GB sponge is an efficient sorbent for crude oils, with high sorption capacity reaching 85–95 times its weight. Furthermore, the authors stated that the crude-oil-sorption capacity of the synthesized GB sponge was remarkably higher than that of the commercial sheet by almost five times. Hence, the GB sponge has a great potential in marine spilled-oil removal and hydrophobic solvent removal [88]. Yang et al. [91] reported a cost-effective, environmentally friendly, and mild approach to fabricate graphene-based sponge (GS) using in situ reduction-assembly of graphene sheets on the melamine sponge skeletons. The hydrophobic and oleophilic GS fabricated in this study exhibited high absorption capacities for oils and organic liquids with excellent recyclability. These superb performances make the GS an effective candidate for potential applications in oil/water separation [109]. Peng et al. [110] reported a cost-effective and facile method to synthesize a novel, superhydrophobic, and robust kaolinite modified graphene oxide-melamine sponge (K-GOMS). The graphene oxide (GO) sheets were used by the authors to enhance the roughness of the sponge surface. Several oils and organic solvents were selected by the authors to test the adsorption performance of the adsorbents. The results of the study demonstrated that the superhydrophobic K-GOMS exhibited a superior adsorption capacity for several types of oils and organic solvents. Hence, this study provides an effective approach for fabricating low-cost, facile, and large-scale production of superhydrophobic adsorbents for oil/water separation [110].

Nowadays, oil spills are considered a serious worldwide environmental crisis. To deal with this global issue, Meng et al. [111] synthesized a super-lipophilic and super-hydrophobic functionalized graphene oxide/polyurethane (FGP) sponge via a simple and cost-effective dip coating method. The synthesized FGP sponge by the authors demonstrated a great absorption capacity for a wide variety of oils and organic solvents from water, reaching 35 times its own mass. In addition, the FGP sponge demonstrated a good reusability. Therefore, this study provides a simple approach for the removal of oil spills and organic solvents from water [111]. Sun et al. [52] mentioned that modifying the commercial melamine sponge (MS) with graphene oxide (GO) or GO-based nanocomposites (NCs) is a facile, efficient, and low-cost method to synthesize three-dimensional (3D) porous materials with multifunctionality. Sun et al. [52] synthesized two types of 3D MSs that were separately functionalized via the reduced GO (RGO) sheets and Ag/RGO NC using combined methods of immersion process and chemical reduction. The results of the study demonstrated that, owing to the high porosity and unique surface topography, the RGO-MS exhibited a high absorption capacity, reaching 41 to 91 times its own weight for several types of oils and organic solvents. Furthermore, both the Ag/RGO-MS and RGO-MS showed high oil/water separation efficiencies. Thus, Ag/RGO-MS and RGO-MS are very promising candidates for potential applications in oil-spill removal and water disinfection [52]. Zhang [98] proposed a facile method to synthesize superhydrophobic polyurethane sponge for oil/water separation using a simple dipping-drying process. The results of the authors’ study showed that the GSH-based superhydrophobic sponge exhibited high absorption selectivity for a wide range of oils and organic solvents. This study demonstrated a new effective way to synthesize superhydrophobic materials for oil/water separation, with a great potential in large-scale industrial practical application [98]. Periasamy et al. [86] synthesized polyurethane dish-washing (PU-DW) sponges functionalized sequentially via polyethylenimine (PEI) and graphene oxide (GO) producing PEI/reduced graphene oxide (RGO) PU-DW sponges. The PEI/RGO PU-DW sponge was coated with 20% phenyltrimethoxysilane (PTMOS) to further enhance the absorption capacity and hydrophobicity of oil. The results of the authors’ study showed that the PTMOS/PEI/RGO PU-DW sponge absorbed a wide range of oils within 20 s and efficiently separated oil/water mixtures through a flowing system. Thus, with the merits of a fast absorption rate, low cost, and reusability, the PTMOS/PEI/RGO PU-DW sponge has a great potential as a superabsorbent for highly efficient removal and recovery of oil spills as well as for the separation of oil/water mixtures [86]. Zhou et al. [112] synthesized graphene/polyurethane (PU) sponges with superhydrophobicity via the one-pot solvothermal technique. The synthesized graphene/polyurethane (PU) sponge can act as a selective filter to effectively and continuously separate oil from water. Owing to the functionalized PU sponge’s structural integrity, the material was capable of separating oil up to 53,000 times its own mass with an oil–water separation efficiency greater than 99.5%. This study shows that the functionalized PU sponge and the integrated oil–water separation system provided have a great potential in industrial scale oil–water separation processes [112]. Zhao et al. [113] fabricated graphene-coated melamine sponges (GCMSs) via a one-step method. The GCMSs synthesized by the authors are hydrophobic, oleophilic, and demonstrated high absorption for oil liquids. The results of the study showed that the absorption capacities for diesel oil and gasoline were greater than 105 g g^−1^. Thus, GCMS has great application potential in the field of oil spillage cleanup [113]. Yang et al. [56] synthesized a hydrophobic and superoleophilic nitrogen-doped graphene (NG) sponge with a three-dimensional (3D) structure via a facile hydrothermal method without further surface modification or pretreatments. The as-prepared NG by the authors exhibited a good porosity, low density, and high specific surface area. The results of the study show that the NG sponges have strong absorption capacities, reaching 200 times its own weight in a short time for a wide range of oils and organic solvents. Thus, this study demonstrates that the NG sponge is a promising candidate in the field of spilled oil recovery owing to its feasibility, cost-effectiveness, and scalability [56]. In addition, Wu et al. [101] mentioned that the graphene sponge (GS) has many applications in oil removal owing to the hydrophobic nature of graphene sheets. However, current hydrothermal preparations of GS require the application of toxic reducing reagents that cause environmental pollution. Hence, several studies focused their work on finding an environmentally friendly methods to synthesize graphene sponge (GS). Wu et al. [101] reported that graphene oxide (GO) can be hydrothermally reduced by glucose forming GS for the adsorption of various oils and organic solvents. The authors have reduced the graphene sheets by glucose during the hydrothermal treatment and produced a 3D porous structure. The study results demonstrated that GSs have efficiently adsorbed oils and organic solvents with competitive adsorption capacities. Thus, GSs can be efficiently used for the remediation of oils from water [101].

### 3.2. Graphene-Based Hydrogels for Oil/Water Separation

Graphene-based hydrogel materials have gained great attention in several fields and applications, including oil/water separation. Various studies have focused their work on using graphene-based hydrogels for oil/water separation. Hu et al. [114] reported a novel, facile, cost-effective, and environmentally friendly method to synthesize fluorographene nanosheets via Michael’s addition reaction and the synthesis of amphiphobic LA/F/rGO hydrogel, based on a hydrothermal process of partially reduced graphene oxide (rGO) at low-temperature and 1H,1H,2H,2H-perfluorodecanethiol (PF) in the presence of l-ascorbic acid (LA) as a reducing agent. The results of the study show that the as-prepared LA/F/rGO hydrogel demonstrated oil bouncing and intriguing oil repellant behaviors underwater. The authors also added that pre-soaking with oil or water allows selective and efficient separation of oil or water from their mixtures. Thus, the new LA/F/rGO hydrogel synthesized in this work has proven to be a promising candidate in oil/water separation, wastewater treatment, and oil fence material. Hence, this study provides new approaches to the fabrication of novel graphene-based nanocomposite materials for oil/water separation processes and oil leaking, with various environmental and engineering applications [114]. The continuous increase in the production of produced water from oilfields is proven to cause detrimental environmental effects. Fong et al. [115] fabricated an efficient, environmentally friendly, and recyclable reduced graphene oxide immobilized κ-Carrageenan hydrogel composite (κCaGO) as an alternative sorbent for crude oil-in-water demulsification. Polyethyleneimine (PEI) was employed by the authors to produce a stable hydrogel composite. The immobilization conditions of graphene oxide (GO) on PEI-modified κ-Carrageenan (κC) beads were appropriately optimized by the authors. The results of this study show that the synthesized κCaGO could be efficiently used as a potential sorbent substitute for the separation of crude oil from produced water [115]. Sun et al. [116] presented a simple droplet microfluidic method for generating graphene oxide (GO) hydrogel composite particles for oil decontamination. The synthesized GO hydrogel composite particles provided in this study have proven to be an ideal candidate for oil decontamination [116]. The addition of graphene with its unique properties is shown to enhance the performance of traditional polymer hydrogels. Graphene in hydrogels acts as a gelator to self-assemble into the hydrogels. In addition, as a filler, it blends with small molecules and macromolecules, resulting in the production of multifunctional hydrogels. The addition of graphene to the hydrogels enhances its electrochemical performance and leaves the door open for a large number of applications in addition to oil/water separation. Such applications can extend to other water treatment applications (dye and metal removal), medical treatments, biomedical, tissue engineering, supercapacitor, and many others. Most of the published works on hydrogels are theoretical and applied at a small scale. A proper large-scale application and effective demonstration that exploits the full potential of graphene hydrogels is still lacking. The design and fabrication of new graphene hydrogels and the effect of formation mechanisms of the graphene and gels are not fully understood, nor researched.

### 3.3. Graphene-Based Aerogels for Oil/Water Separation

Graphene aerogels are the world’s lightest materials, with an unusual low density [117]. The combination of the hydrophobic properties of graphene sheets and low density makes the graphene aerogel a very promising candidate for oil absorption. The absorption capacity of the graphene aerogel approaches a level several hundred times greater than that of commercially available materials for environmental remediation purposes. Nowadays, using three-dimensional graphene aerogel materials for oily wastewater treatment with its complex compositions remains a great challenge owing to volume shrinkage, which results in low adsorption capacity and single-function. Ji et al. [118] introduced renewable Enteromorpha into the graphene aerogel using facile hydrothermal-freeze casting treatment, producing the ultralight, compression, and amphiphilic adsorbent for oil spill removal and water pollution cleanup. The results of this study show that, for clean-up of oil spills, the Enteromorpha modified graphene aerogel (EGA) showed superb adsorption capacity towards oil and organic solvents compared with pristine graphene aerogel (GA). Hence, EGA can be successfully used as an adsorbent for oil in oil/water separation processes [118]. Chatterjee et al. [119] developed a simple preparation method to synthesize aerogels with great shape retention for oil absorption application. To increase the selective oil absorption and hydrophobicity, the authors used a dip coating process to chemically modify the graphene sheets and attach it to the aerogel pore surface. Several examples were demonstrated by the authors to show the feasibility of applying the synthesized aerogel in oil spill control [119]. Zhan et al. [120] investigated the role of graphene oxide (GO) in increasing the oil absorption capacity by the aerogel materials and in decreasing the shrinkage in polyimide (PI) aerogels. The study includes grafting the GO particles with pyromellitic dianhydride (PMDA) forming GO-modified PMDA prior to reaction with 2,2′-dimethylbenzidine to form polyamic acid. The polyamic acid chains are then cross-linked using 1, 3, 5-tris (4-aminophenyl) benzene and chemically imidized using acetic anhydride and pyridine to obtain polyimide gel. The resultant aerogel specimens, obtained by supercritical drying of the gels, demonstrated great reduction of diameter shrinkage from 9.0% for unmodified monoliths to 0.8% in the presence of 5.2 wt% GO, a 9.1% reduction in surface energy compared with the unmodified aerogel, high surface area (>504 m^2^/g), high porosity (>93%), and low bulk density (<0.0905 g/cm^3^). These aerogels demonstrate a great increase in the hydrocarbon oil absorption capacity [120]. Meng et al. [121] synthesized a lignin-based carbon aerogel enhanced by graphene oxide (LCAGO) for the separation of oil from water. The synthesized aerogel by the authors demonstrated superhydrophobicity and good mechanical property, proving that it is capable of being used in oil/water separation. The results of the authors’ study reveal that the aerogel efficiently separated the light oil, heavy oil, and emulsified oil from water. The results of the study indicate that the material is highly effective and very practical as a water-cleaning material. In addition, the preparation process provided by the authors is energy-saving and is an efficient route for the separation and collection of oil from water with industry waste [121].

Recently, graphene aerogels have gained great research attention in oil/water separation processes based on their distinguishable properties. However, the over stacking of graphene oxide nanosheets (GO) results in a poor recyclability and low adsorption capacity. Kang, Cui [51] fabricated nitrogen-doped magnetic carbon nanospheres/graphene composite aerogels (MCNS/NGA) under weakly alkaline conditions via a one-step hydrothermal in situ electrostatic self-assembling strategy. The aerogels synthesized by the authors had super-elasticity, low density, high specific surface area, and good magnetic properties. Hence, the aerogels exhibited high adsorption capacity in the range of 187 g g^−1^ to 537 g g^−1^ towards various oils and organic solvents, surpassing most of the reported materials to date. Thus, the MCNS/NGA synthesized in this study are promising candidates in oil/water separation processes [51]. Cleaning up of crude-oil spills is considered a major environmental remediation issue and urges the fabrication of sorbents with high and stable hydrophobic and oleophilic properties. Thakkar, Pinna [122] synthesized aerogels with durable hydrophobicity via incorporating reduced graphene oxide (rGO) in highly porous silica matrices. The aerogels synthesized by the authors exhibited high oil selectivity and sorption. Hence, the aerogels fabricated in this study can be successfully implemented in oil/water remediation processes [122].

Low fluidity and high viscosity heavy crude oils frequently decrease their diffusion rate into the porous adsorbents, leading to a low efficiency in oil spill removal. Hence, the photothermal effect can be used to greatly decrease the viscosity of heavy crude oil via in situ solar light heating. Luo, Wang [123] synthesized photothermal carbon nanotube/reduced graphene oxide (CNT/RGO) microspherical aerogels by the fabrication of graphene oxide (GO)-based microspherical aerogels with several radially orientated microchannels, followed by high-temperature reduction of the GO components and growing CNTs inside the microchannels. The results of the study show that, under 1 sun irradiation, the aerogel surface temperature quickly rose to 83 °C in 1 min, causing a rapid decrease in the viscosity of crude oil. Moreover, the optimal microspherical aerogels exhibited an excellent adsorption capacity of heavy crude oil, up to 267 g g^−1^ in only 10 min, surpassing several other reported oil adsorbents [123]. Three-dimensional (3D) aerogels with lipophilic and hydrophobic properties have gained great attention in efficient oil spill clean-up. However, biodegradability, cost-effectiveness, and recycling are still great challenges in the application of aerogels for oil/water separation. Hu, Zhu [124] fabricated high biocompatibility, hydrophobic, and low cost composite aerogels via directional freezing-drying technology with the use of chitosan (CS) as the skeleton substrate, hydrophobic silicon particles/polydimethylsioxane (H-SiO_2_/PDMS) as the hydrophobic modifier, and reduced graphene oxide nanosheets (rGO) as enhancements. The composite aerogel synthesized in this study demonstrated high adsorption capacity for oil, as well as good thermal and chemical stability in a harsh environment. In addition, the adsorbed oils and organic solvents can be easily extruded from aerogels owing to its excellent compressive properties [124]. Cleaning, collecting, and recycling oil from oil spills, specifically high viscosity crude oil, are of worldwide concern. Sun et al. [125] synthesized a 3D macrostructure CuFeSe_2_-loaded graphene aerogel (GA-CuFeSe_2_) that shows an outstanding photothermal conversion capacity, surpassing various other materials. The synthesized 3D macrostructures supported by graphene holds a versatile pore structure that allows rapid diffusion of highly viscous oils and a high corrosion resistance. The high-performance oil sorption and photothermal conversion, driven by solar power, demonstrated that this material can be efficiently applied for oil spill clean-up [125]. Zhang et al. [106] developed a facile approach for a highly efficient oil/water separation process by incorporating dimethyldiallylammonium chloride acrylamide polymer (P(AM-DMDAAC)) into the graphene aerogels. The functionalized 3D graphene aerogel presented several outstanding physical properties, including low density, large specific surface area, and great hydrophobicity. The modified aerogel in this study demonstrated excellent adsorption capacity for a wide range of oils and organic solvents. The authors have also found a simple approach by changing the pH values to achieve controlled wettability transition of P(AM-DMDAAC)/graphene aerogels (PGAs). The hydrophobic PGA prepared at pH 2.03 demonstrated a superb oil/water separation performance. Thus, as an efficient and recyclable water purification material, the environmentally friendly and sustainable polymer-modified graphene aerogel has promising potential in oil/water separation [126].

In a severely damaged marine polluted environment, low-density and environmentally friendly aerogels have become potential materials for oil/water separation. However, several reported aerogels have the disadvantages of low oil absorption, poor flexibility, and compressibility, which hinders their application. Zhou et al. [127] reported an anisotropic, compressible lamellar lipophilic and hydrophobic graphene/polyvinyl alcohol/cellulose nanofiber carbon aerogel (a-GPCCA) prepared via carbonization and directional freeze-drying processes. The synthesized synthetic ultralight a-GPCCA had high porosity, reaching 99.61%, and low density (6.17 mg/cm^3^). In addition, the directional freeze-drying used by the authors produced a lamellar interpenetrated three-dimensional (3D) porous structure, with a high adsorption capacity, good compressibility, and recyclability. Moreover, carbonization provided it with a high thermal stability and hydrophobic properties, resulting in a high oil/water selectivity and combustion cyclicity. Thus, the a-GPCCA fabricated in this study has promising potential in the field of the offshore oil spill treatment and domestic industrial wastewater [127]. Song et al. [128] have successfully synthesized a grass-modified graphene aerogel (GGA) using an environmentally friendly, simple, and low-cost hydrothermal treatment. The graphene aerogel properties have been significantly enhanced by introducing the grass powder, leading to a highly efficient selective adsorption process. The results of this study show that the GGA can successfully be used in the oil/water separation of mixed solution via different manipulation methods. In addition, the GGA is capable of removing oils via adsorption, acting as a filter media dealing with oil/water mixtures, and collecting oils continuously using a peristaltic pump or gravity. This study demonstrates that the GGA is a very good and promising candidate in the oil/water separation field and spill oil clean-up for environmental remediation. Moreover, the addition of grass enhances the graphene aerogel’s oil adsorption efficiency, hydrophobicity, and mechanical property, providing a new route for high-value utilization of biomass waste [128]. Huang et al. [117] synthesized cork-like graphene aerogels via the EDA-ammonia double hydrothermal reduction method. The fabricated porous aerogels demonstrated high hydrophobicity, porosity, and excellent mechanical properties. In addition, the oil adsorption efficiency is significantly greater than that of the conventional adsorbents, with an oil adsorption capacity of 130.10 g/g for pure diesel and continuous treatment capacity of 71.67 g/g for emulsified oil in oily contaminated water. Hence, the continuous aerogel-based water treatment process is proposed in this study for graphene-based aerogels to have promising potential applications in oil spill remediation and water purification [117]. Chen, Li [129] have facilely modified an ultralight, compressive, and fire-resistant graphene aerogel via renewable lignin biomass with porous and hydrophobic skeletons. The lignin-modified graphene aerogel (LGA) in this study showed a highly efficient absorption of petroleum oils. Hence, LGA is a highly efficient, renewable, and recyclable potential candidate for applications in oil/water separation [129].

Low-density aerogels are very effective materials for oil absorption; however, they are limited by their low elasticity and compressibility. Mi et al. [130] prepared three-dimensional (3D), highly compressible, anisotropic, elastic, cellulose/graphene aerogels (CGAs) via bidirectional freeze-drying. The authors have grafted long carbon chains using the chemical vapor deposition method, forming modified cellulose/graphene aerogels (MCGA) with an enhanced superhydrophobicity. Thanks to its ultra-light weight and high surface area, MCGA holds an excellent absorption capacity of 80 to 197 times its weight, which surpasses most carbon-based aerogels and hydrophobic cellulose aerogels. Thus, MCGA is a very promising absorbent material for selective oil absorption and recovery [130]. In recent years, reduced graphene oxide (rGO) has attracted great attention from researchers around the world owing to its outstanding physicochemical properties and distinguishable surface chemistry. Cao et al. [131] developed a facile and environmentally friendly method to fabricate reduced graphene oxide/polydopamine composite aerogel modified by 1*H*,1*H*,2*H*,2*H*-perfluorodecanethiol (PFDT) and reinforced by chitosan. The aerogel synthesized by the author demonstrated high oil/water separation behavior, showing that the aerogels are very effective materials for the remediation of water from oil spill accidents [131]. Yu et al. [132] developed ultralight and nitrogen-doped graphene aerogels (UNGAS) via a hydrothermal method in the presence of graphene, L-arginine, and dopamine (DA). The synthesized UNGAS demonstrated high absorption capacity for several oils owing to low density, large surface area, and N-doping [132]. Cheng et al. [133] reported the preparation of a new type of ultralight 3D porous and highly hydrophobic graphene aerogel (GA) with melamine formaldehyde (MF) microspheres as a spacer and pore forming agent and graphene oxide as a precursor, followed by freeze-drying, hydrothermal reduction, and calcination process. The resultant calcined MF-GA (cMF-GA) exhibited a fast absorption rate and maximum sorption capacities up to 230 g/g for diesel oil. Thus, cMF-GA is a promising highly efficient and low-cost absorbent in practical applications of large-scale oil removal [133]. Kabiri et al. [134] reported a green method for fabricating graphene–carbon nanotube aerogels with three dimensional (3D) interconnected networks formed by natural graphite rocks. CNTs are then incorporated by the authors into the network to enhance the hydrophobicity and robustness of aerogels with excellent oleophilic properties and porosity. The aerogels prepared in this study demonstrated an outstanding adsorption for petroleum products, specifically under continuous vacuum regime, with an adsorption capacity reaching 28 L of oil per gram of aerogel. The given synthetic method in this study is economical and simple for the scalable fabrication of highly porous 3D graphene–carbon nanotube aerogels, which can be efficiently used for an effective and low cost oil spill clean-up for oily water purification [134]. Hong et al. [135] synthesized a functionalized graphene aerogel with high hydrophobicity and porosity via surface modification of self-assembled graphene oxide aerogels. The functionalized graphene aerogel demonstrated remarkable physical features, including low density, high porosity, mechanical stability, and hydrophobicity. The functionalized graphene aerogel in this study exhibited excellent absorption performance for several types of oils and organic solvents, making them a promising candidate for oil spill clean-up [135].

### 3.4. Graphene Foam

Oil leakage accidents usually occur during the production, transportation, and application of petroleum products, which is a common and disastrous environmental issue. It is of great importance and very challenging to develop efficient oil/water separation materials. Hence, several studies are conducted to tackle the oil/water separation issue. Liu et al. [136] reported a feasible and simple method to fabricate high-performance and efficient 3D graphene foam (GF) oil-absorbing material. The authors have loaded gold nanoparticles (Au NPs) on the surface of graphene foam via ion sputtering and then modified the surface with 1H, 1H, 2H, 2H-perfluorodecanethiol (PFDT). The prepared graphene foam by the authors is porous with an excellent water repellency and large specific surface area. The superhydrophobicity demonstrated by the materials is attributable to the interaction between the reduction of surface energy by PFDT and the rough structure of gold nanoparticles. These superb properties allow the functionalized graphene foam to have an outstanding oil absorption capacity, reaching 25.8 g/g. Thus, the prepared graphene foam has great potential as an efficient absorbent material for oil spill purification [136]. Krebsz et al. [137] presented the fabrication of bio-graphene foams (bGFs) using renewable sources, and the application of bGFs as a new class of adsorbents for the removal of oil contaminants from waste water and chromate ions. The bGFs synthesized by the authors showed an excellent performance in the separation of various types of oils from water in a continuous oil/water separation process, showing 98.8% separation efficiency for petroleum. Consequently, this study confirms that the bGFs are efficient adsorbents for oil/water separation processes [137]. Jo et al. [138] reported the preparation of robust superoleophilic and superhydrophobic MoS_2_ nanoparticles and reduced graphene oxide (rGO) incorporated polyurethane (PU) foam via in situ polymerization using the one-shot method. The oil absorption capacity of the foam was examined by the authors via standard sorption testing. The modified system demonstrated very high oil selectivity, making it an efficient material for oil/water separation processes [138]. Lui et al. [139] prepared superoleophilic and superhydrophobic graphene-melamine foam (rGO-MF) via a simple soaking and chemical reduction process. The rGO-MF exhibited a porous and repetitively compressible structure, with an excellent absorption capacity for several types of oils. Thus, the rGO-MF has significant potentials in the field of oil/water separation and oil absorption [139].

The fabrication of cost-effective and robust adsorbents for quick and easy clean-up of oil spills is highly required. Lv et al. [140] synthesized one novel porous melamine foam (MF) with both hydrophobic magnetic nanoparticles and reduced graphene oxide (rGO) decorated on its skeleton (r-MGMF) using a reductive annealing process and stepwise assembly. The superhydrophobic surface, porous structure, and magnetic property of r-MGMF made it highly efficient in the separation of oil from water with an excellent oil adsorption capacity. The results of the study show that the r-MGMF is competent in the continuous separation of oil/water mixed flow, with a separation efficiency greater than 96.33%. Thus, the hydrophobic stability, high selectivity, excellent recyclability, and large adsorption capacity prove that the r-MGMF is a great potential material in oily wastewater treatment [140]. Yang et al. [141] reported the fabrication of magnetic graphene foam incorporated with magnetite (Fe_3_O_4_) nanoparticles and its application for the adsorption of several oils and organic solvents. The fabricated foam exhibited an outstanding oil adsorption capacity, excellent stability, and recyclability under cyclic operation and high restoration for absorbates. Hence, the simple and effective method presented in this study for the preparation of graphene foams offers a new route for scaled-up production of graphene materials applied in the clean-up of oil spills [141].

Superoleophilic and superhydrophobic foam-like materials are gaining great attention as a promising absorbent material for oil spill clean-up from oily contaminated water. Oribayo, Feng [7] reported the development of lignin-based polyurethane (LPU) foam and its surface modification, forming a superoleophilic and superhydrophobic sorbent, for use in spill clean-ups. The authors have grafted the interior matrix of the LPU foam with adhesive octadecylamine (ODA) and polydopamine-reduced graphene oxide (rGO), which enhanced the LPU foam skeleton to a superoleophilic and superhydrophobic 3D structure. The results of the authors’ sorption experiments revealed that crude oil, kerosene, engine oil, and chloroform were efficiently absorbed by the LPU-rGO-ODA foam with a sorption capacity of 26–68 times its own weight. In addition, the LPU-rGO-ODA foam exhibited a high selectivity to oil sorption and superb reusability over several sorption-squeezing cycles. Hence, LPU-rGO-ODA foam is expected to be a very promising oil sorbent material for potential applications in oil spill remediation [7]. Liu et al. [87] fabricated a magnetic polymer-based graphene foam (MPG) for oil–water separation processes using the synergistic effects of the Fe_3_O_4_ nanoparticles deposition on graphene sheets and the self-assembly of graphene on polyurethane (PU) sponge. The resulting MPG demonstrated high superoleophilicity and superhydrophobicity with an oil contact angle of 0° and water contact angle of 158 ± 1°. In addition, a wide range of oils and organic solvents were removed from oil–water mixtures under manipulation using a magnet bar with high selectivity and absorption capacity. Therefore, the results of this study offered a facile method for the clean-up of crude oil polluted water and the removal toxic organic solvents [87]. Song et al. [128] reported the synthesis of stable, cost-effective, and recyclable superhydrophobic reduced graphene oxide modified melamine foam (RGMF) using an ultrasonic-microwave synergistic method for the first time. The synthesized RGMF demonstrated an excellent selective adsorption capacity for several types of oils and organic solvents from water. In addition, the maximum oil adsorption capacity attained by the foam was 112 times its initial weight. Consequently, all these properties made the as-prepared material in this study an ideal candidate for the removal of various oils and organic solvents from water [142]. All the potential advantages and the use of GO-enhanced foam application lead to the potential use of low voltage applications for the separation of viscous oils from water.

### 3.5. Graphene Decorated Meshes for Oil–Water Separation

**Figure 5:**
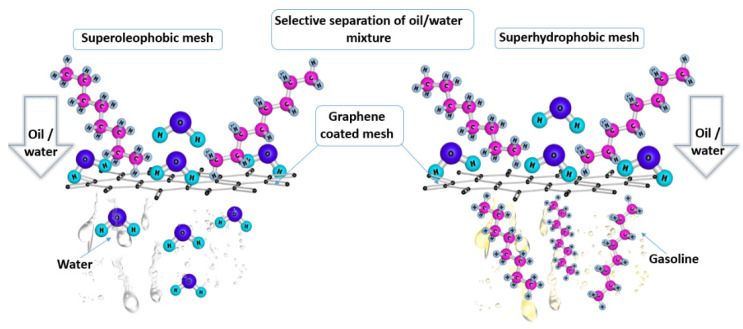
Selective separation of an oil/water mixture (gasoline and water) using a graphene-coated mesh.

Synthesizing underwater superoleophobic filtration materials with excellent anti-oil-fouling performance and robust stability in harsh environments is required for high efficiency oil/water separation processes. Graphene-coated meshes are a very promising candidate for oil/water separation processes, with its oleophobicity, hydrophobicity, and simple separation mechanism (Figure 5). Chen et al. [143] adopted irregular hydrophilic graphene oxide (GO) as a coating material to modify an oxidized copper mesh with the desired hydrophilic composition and hierarchical surface roughness using a novel in situ copper ion induced crosslinking approach. The as-prepared GO@CuO mesh by the authors was efficiently applied in the separation of oil/water mixtures, with an efficiency greater than 99.49% and good reusability. As a result of the high oil–water separation efficiency, excellent anti-oil-fouling property, and good stability, the prepared underwater superoleophobic mesh in this study is an ideal effective candidate for broad applications in oil/water separations [143]. Lu et al. [44] reported a mild, environmentally friendly, and cost-effective method to synthesize graphene oxide (GO) and reduced graphene oxide (RGO) coated meshes with superoleophobicity and superhydrophilicity/underwater. The GO-coated meshes showed a high unidirectional separation efficiency for water mixtures with various light oils, while the RGO-coated meshes exhibited an outstanding capability of separating several types of heavy oils from water. The results of this study show that both types of meshes could attain their original wettability in harsh environments, and demonstrate high recyclability and efficiency for the separation of oil/water mixtures containing boiling water, salt, and acid. [44]. Yin et al. [144] successfully fabricated an environmentally friendly stainless steel mesh (SSM) decorated with graphene oxide (GO) and polydopamine (PDA) for oil/water mixture separation using a facile dipping coating method. The as-prepared SSM/PDA/GO mesh in this study was capable of separating oil/water mixtures with an ultra-high flux reaching 15,000 L × m^−2^ × h^−1^ and an outstanding separation efficiency, which is greater than 99.95%. Moreover, SSM/PDA/GO possesses an outstanding stability under harsh environmental conditions, indicating that the mesh is a promising candidate material for practical oil spill remediation in harsh conditions, specifically in a highly salty marine environment [144].

### 3.6. Graphene-Coated Cotton for Oil/Water Separation

The development of functional materials that can separate oil from water in an energy efficient way is highly required, but yet still challenging. Graphene-coated cotton fabrics provide an efficiently clean way of removing oils from water. Yang et al. [145] demonstrated a mild, environmentally friendly, and low-cost method to synthesize underwater superoleophobic graphene oxide (GO) coated fabric via dip-coating of GO nanosheets onto the cotton fabric. In addition, the superhydrophilic GO coated fabric can be transformed into a superhydrophobic reduced graphene oxide (rGO) coated fabric by chemical reduction of the coated GO fabric. The GO and rGO coated fabrics demonstrated outstanding efficiencies for unidirectional separation of several light and heavy oils from water. Moreover, both types of fabrics showed high efficiency and recyclability for the separation of oil/water mixtures containing acid, hot water, and salt. Thus, the synthesized fabrics are potentially efficient candidates for the separation of various oil/water mixtures [145]. Ge et al. [146] fabricated a graphene-coated cotton via a self-assemble technique. This graphene-coated cotton has efficiently absorbed various types of oils, owing to its superoleophilicity and superhydrophobicity. Thus, this study demonstrates that the graphene-coated cotton is a promising candidate for the application in large-scale removal of oils from water [146]. Hoai et al. [147] prepared a reduced-graphene-oxide (RGO)-coated cotton sponge (RGO-Cot) via simply heating a graphene-oxide (GO)-coated cotton sponge. The prepared RGO-Cot sponge exhibited superoleophilicity and superhydrophobicity, with a water contact angle of 151°. Hence, these RGO-Cot sponges can be used efficiently for the removal of various types of oils and organic solvents, as they exhibit high absorption capacities and good absorption recyclability [147].

## 4. Current Challenges and Future Perspectives

Graphene and its derivatives are gaining attention in many applications including oil/water separations. Although reports show great advancements and potential applications in oil/water separations, this technology still faces a number of challenges that need to be overcome. The issues are mainly related to the industrial production and effectiveness. One of the main challenges is the level of attachment of graphene and GO onto the support material, as it can easily detach from the substrate during operation. This can be attributed to the weak van der Waals forces, which is the main force binding the GO to its host. Accordingly, advancing the preparation methods and possible addition of various adhesives are indeed of importance. In the case of using sponges and aerogels, the movements and collection of the sponges and aerogels are required to effectively apply them for the offshore oil/water spills. The use of magnetic materials has been reported. In addition, further considerations of the effectiveness of the system in salted water (such as in seawater oil spill applications) and sweet water applications are needed to fully understand the applicability of the systems. Furthermore, multiphase oil/water separations need to be considered as most studies focus on experimental single-phase oil/water mixtures. A final remark is related to the scale up of the process. Up-to-date, all studies have been focusing only on laboratory small-scale systems and no studies have been conducted on larger scale studies. Scaling up studies are required to test the effectiveness of the system, as well as its applicability at higher flow rates and under conditions that are more realistic. Although, recent advancements in graphene-related sponges are still considered fragile and lack mechanical and chemical strength. Accordingly, advancements related to enhancing and improvements of inter-sheet binging are needed. To this end, the use of surface modification, optimization of crosslinking agents, and addition of highly elastic polymers might offer improvements in their application.

## 5. Conclusions

Graphene is a versatile material that has many potential applications in its pristine and derived constituents in oil/water separation applications. The graphene-based technologies discussed include the following graphene-based forms: membranes, sponges, hydrogels, aerogels, foams, and graphene-coated cotton and decorated meshes. The review discussed the recent advancements in graphene and graphene-based materials in those applications. The advantages and shortcomings in addition to the future challenges and perspectives were addressed. The review highlighted that graphene and its derivatives will continue to make a significant contribution to the advancement of oil/water separation.

## Figures and Tables

**Table 1:** Physical properties of graphene.

Physical Property	Value	Reference
Charge carrier mobility	200,000 cm^2^/V·s	[57]
Thermal conductivity	5000 W/m·K	[58]
C–C bond length	1.4 2 Å	[59]
Specific surface area	2630 m^2^/g	[60]
Optical transparency	97.7%	[61]
Tensile strength	1100 GPa	[62]
Young’s modulus	2.4 ± 0.4 TPa	[63]
Resistivity	10^−6^ Ω cm^2^	[64]
Band gap	Zero	[65]

**Table 2:** Oil rejection efficiency of some graphene-based membranes in several oil/water mixtures.

Membrane	Feed Components	Operating Pressure (MPa)	Oil Rejection (%)	Oil/Water Flux (L·m^−2^·h^−1^·bar)	Ref.
Cardanol-GO	Petroleum ether/water containing CuSO_4_	Gravity driven	99%	N/A	[68]
PGS/GO	pump oil, hexadecane, soybean oil/ultrapure water	0–0.15	>99.9%	3734	[69]
TiO_2_/SGO/Ag	Gasoline, toluene, n-heptane, chloroform/water	Gravity driven under UV	99.6%	53–175	[70]
PEI-g-GO	Water/hexane	Gravity driven	99%	688	[71]
SiO_2_/GO	Soybean, gas, diesel, pump oil/DI water	Vacuum (~0.1)	99.4%	470	[72]
GO-ePOSS	CH_2_Cl_2_, petroleum/H_2_O	Gravity driven	99%	N/A	[73]

**Table 3:** Sorption capacities of several graphene-based modified sponges for oil/water mixtures.

Sorbent Material	Type of Oil	Sorption Capacity (g/g)	Reference
rGO@MF modified sponge	Crude oil-in-water	2.1–5.6	[92]
Graphene-based sponge	Oil and organic solvents	50–165	[93]
SiO_2_/GO-PU sponge	Oil and organic solvents	80.0–180.0	[94]
Graphene-coated PU sponge	lubricate oil	31.0	[95]
NMP/graphene PU sponge	Oil and organic solvents	40.0–80.0	[96]
GO/PU sponge	Oil and organic solvents	30.0–55.0	[97]
Thiolated graphene/PU sponge	crude oil	29.5–90.0	[98]
RGO/PU sponge	Oil and organic solvents	24.2–37.6	[99]
RGO/OAP/PU sponge	Oil and organic solvents	24.7–80.3	[100]
G sponge	Machine oil	35.5	[101]
Polyethylenimine/RGO decorated PU sponge	Bicycle chain oil	8.8	[86]
RGO and octadecylamine decorated PU sponge	Silicon oil	29.7	[7]

## Data Availability

Not applicable.
